# Association analysis of physiological traits in spring barley (*Hordeum vulgare* L.) under water‐deficit conditions

**DOI:** 10.1002/fsn3.2161

**Published:** 2021-01-28

**Authors:** Mitra Jabbari, Barat Ali Fakheri, Reza Aghnoum, Reza Darvishzadeh, Nafiseh Mahdi Nezhad, Reza Ataei, Zahra Koochakpour, Mitra Razi

**Affiliations:** ^1^ Faculty of Agriculture Higher Education Complex of Saravan Saravan Sistan and Baluchestan Iran; ^2^ Department of Plant Breeding and Biotechnology Faculty of Agriculture University of Zabol Zabol Sistan and Baluchestan Iran; ^3^ Seed and Plant Improvement Research Department Khorasan Razavi Agricultural and Natural Resources Research and Education Center AREEO Mashhad Khorasan Razavi Iran; ^4^ Department of Plant Production and Genetics Faculty of Agriculture and Natural Resources Urmia University Urmia Iran; ^5^ Seed and Plant Improvement Institute Agricultural Research, Education and Extension Organization (AREEO) Karaj Iran; ^6^ Department of Plant Production and Genetics Faculty of Agriculture and Natural Resources Urmia University Urmia Iran

**Keywords:** barley, drought, linkage disequilibrium, mixed linear model, physiological traits

## Abstract

In the present study, 148 commercial barley cultivars were assessed by 14 AFLP primer combinations and 32 SSRs primer pairs. Population structure, linkage disequilibrium, and genomic regions associated with physiological traits under drought stress were investigated. The phenotypic results showed a high level of diversity between studied cultivars. The studied barley cultivars were divided into two subgroups. Linkage disequilibrium analysis revealed that *r* ^2^ values among all possible marker pairs have an average value of 0.0178. The mixed linear model procedure showed that totally, 207 loci had a significant association with investigated traits. 120 QTLs out of 207 were detected for traits under normal conditions, and 90 QTLs were detected for traits under drought stress conditions. Identified QTLs after validation and transferring to SCAR markers in the case of AFLPs can be used to develop MAS strategies for barley breeding programs. Some common markers were identified for a particular trait or some traits across normal and drought stress conditions. These markers show low interaction with environmental conditions (stable markers); therefore, selection by them for a trait under normal conditions will improve the trait value under stress conditions, too.

## Graphical abstract

In the present study, 148 commercial barley cultivars were assessed by 14 AFLP primer combinations and 32 SSRs primer pairs as well as 14 Physiological traits under water deficit conditions. Mixed linear model procedure showed that totally, 207 loci had significant association with investigated traits. Some common markers were identified for a particular trait or some traits across normal and drought stress conditions.These markers show low interaction with environmental conditions (stable markers); therefore, selection by them for a trait under normal conditions will improve the trait value under stress conditions, too.

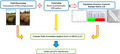

## 1 INTRODUCTION

Barley (*Hordeum vulgare* L.) was domesticated in the Fertile Crescent near 10,000 years ago (Kilian et al., 2006, 2009). It is the fourth most important cereal crop after wheat, rice, and maize (Reddy et al., 2014). Barley yield is reduced by different factors like heat, salt, and drought (Long et al., 2013; Thompson & Woodward, 1994). Among these abiotic stresses, drought has a major effect on plant growth and productivity because plants need water for physiological processes and transporting nutrients and metabolites (Polle et al., 2006). Barley (2n = 2x = 14) is a good genetic model for investigating mechanisms of drought tolerance (Close et al., 2009; Inostroza et al., 2009). In several studies, the effect of drought stress on growth and productivity parameters was investigated in different crops including barley (Barnabas et al., 2008; Farooq et al., 2009). In barley, some parameters like biomass production (Jamieson et al., 1995), yield (Gonzales et al., 1999), photosynthesis rate (Li et al., 2006), free proline accumulation (Sayed et al., 2012), the total content of soluble sugars (Teulat et al., 2001), and osmotic adjustment (Blum, 1989) are affected by drought stress.

Genetic tolerance to drought stress is complex and understanding the genotype, environment and genotype by environment interaction effects are important and vital in designing drought resistant breeding programs (Bänziger & Araus, 2007; Yue et al., 2006). Thanks to the development of molecular markers technology, it is now possible to decipher the genetics of complex traits and identify functional genes or markers closely linked to their controlling genes (Pinto et al., 2010). Genome‐wide association study (GWAS) is a suitable method for identifying molecular markers associated with the genomic region involved in controlling complex traits such as drought tolerance (Kazemi et al., 2020; Yan et al., 2011). Association analysis is a method in the search for detecting relationships between phenotypic diversity and genetic polymorphisms in natural populations (Remington et al., 2001; Simko, et al., 2004; Simko, Haynes, et al., 2004; Thornsberry et al., 2001; Wilson et al., 2004). This method has been used in different plant crops, including maize (Thornsberry et al., 2001), barley (Kraakman et al., 2006), wheat (Breseghello & Sorrells, 2006), sunflower (Darvishzadeh et al., 2008; Davar et al., 2012), chickpea (Saeed et al., 2013), and tobacco (Basirnia et al., 2014). GWAS has advantages compared to linkage mapping in identifying QTLs related to traits. It offers higher resolution, do better in identifying QTLs, and save more cost and time (Yu & Buckler, 2006). There are some studies focused on the dissection of drought stress tolerance mechanism in barley (Teulat et al., 1998; Varshney et al., 2012).

As association analysis is based on LD, then the investigation of LD extent in population group is important for successful association analysis (Abdurakhmonov et al., 2008; Sorkheh et al., 2008). In natural populations, LD can be occurred by different factors such as physical linkage, migration, individual relationships, and population structure. Except for physical linkage, the other factors produce LD between loci across different chromosomes, something that is not important in terms of plant breeding. Therefore, population structure and individual relationships are factors that affect the resolution of association analysis and should be considered to avoid false‐positive associations (Bradbury et al., 2007; Zhu et al., 2008). The presence of population structure can be detected by some statistical approaches such as model‐based clustering (Pritchard et al., 2000) and principal component analysis. In the mixed linear model (MLM), Q‐matrix from population structure analysis and the relatedness among individuals are included in the model as covariates to overcome spurious associations between markers and traits (Yu et al., 2006). Association studies in barley have been concentrated on flowering time (Stracke et al., 2009), yield (Gawenda et al., 2015; Kraakman et al., 2004), disease resistance (Massman et al., 2011; Roy et al., 2010), drought tolerance (Varshney et al., 2012; Wójcik‐Jagła et al., 2018), salinity tolerance (Fan et al., 2016), and freezing tolerance (Rapacz et al., 2010; Visioni et al., 2013).

Association analysis can be also conducted based on the candidate genes approach. However, in most studies, a genome‐wide association approach has been used. There are some studies about association mapping based on the analysis of candidate genes, including the *Dwarf8* (Thornsberry et al., 2001) and phytoene synthase locus in maize (Palaisa et al., 2003), flowering time genes in barley (Stracke et al., 2009), the *PsyI‐AI* locus in wheat (Singh et al., 2009), and *rhg‐1* gene in soybean (Li et al., 2009).

The objectives of the present work were to identify population structure in a collection of Iranian barley germplasm genotypes and investigate the association of AFLP and SSR markers with physiological traits in the plant under drought stress conditions. Physiological traits are important in selecting drought tolerance genotypes in plants including barley (Del Pozo et al., 2012), but most of them have quantitative behavior and are controlled by many genes (Mora‐Poblete et al., 2015). Finding QTLs associated with quantitative traits can help breeders to develop a MAS strategy for their breeding programs.

## 2 MATERIALS AND METHODS

### 2.1 Plant material

148 commercial barley cultivars were investigated under normal and drought stress conditions. The seeds of the association panel were received from Khorasan Razavi Agricultural and Natural Resources Research and Education Center. The experiment was carried out at Zahak Agricultural Research Station, Sistan and Baluchistan, Iran (Latitude = 30 Ê 15' N, Longitude = 60 Ê 15' E, Altitude = 480 m).

### 2.2 Experimental design

Genotypes were evaluated using alpha‐lattice design with two replications in well‐watered (irrigation at 90% FC) and drought stress (irrigation at 40% FC) conditions during two successive years. Each replicate includes 11 incomplete blocks with 14 plots, and each replication contains 148 barley cultivars and six local barley varieties (Local, 5‐ White cluster salinity, Nomar, Zahak, NP‐90‐113 and Nimroz). First irrigation was performed for germination, next irrigation was done after the soil moisture reached 90% of the field capacity for well‐watered treatment and drought stress, and irrigation was done after reaching humidity to 40% of field capacity. Moisture measurement was done by Time‐Domain Reflectometer (TDR) method. Physiological traits including canopy temperature (CT), relative water content (RWC), proline content (PRO), water‐soluble carbohydrate concentration (WSC), relative chlorophyll content (RCC), the maximum potential quantum efficiency of PSII photochemistry (Fv/Fm) (PSII), chlorophyll a (Chl a), chlorophyll b (Chl b), the chlorophyll a/b ratio (Chl a/b), carotenoid (Car), catalase (CAT), guaiacol peroxidase (POD), and ascorbate peroxidase (APOX) were evaluated in both years in both irrigation conditions.

### 2.3 Genotyping and population structure

Genotyping of individuals was performed using 14 AFLP primer combinations and 11 SSRs pairs primers (Kraakman et al., 2004, 2006) following the method described by Kraakman et al. (2006). Also, we used 21 new SSR markers from the previously reported map (Aghnoum et al., 2010). Totally, 407 polymorphic markers were used in the present study.

### 2.4 Data analysis

Variance components of phenotypic data were calculated using GenStat version 15 (Payne et al., 2011). Correlation among all studied traits was calculated by SPSS version 24 and heritabilities (Family mean basis) were estimated using SAS version 9.0 software. Best Linear Unbiased Estimates (BLUEs) of phenotypic data based on G × E variances were used in association analysis (Haseneyer et al., 2010). Estimation of the population structure was performed with the Bayesian clustering model (Pritchard et al., 2000) using Structure 2.3. Burn‐in period length was 100,000, and Markov Chain Monte Carlo (MCMC) replications was 100,000. ΔK index was determined for obtaining the optimal subpopulations number, Q‐matrix was derived (Falush et al., 2003; Kraakman et al., 2004; Pasam et al., 2012). The neighbor‐joining dendrogram (NJ) was performed based on the genetic distance matrix using Tassel 5. The linkage disequilibrium (LD) was estimated with Haploview 4.01 software (Barrett et al., 2005). Association analysis was performed using a mixed linear model (MLM) considering Q‐ and K‐matrices (Yu et al., 2006) as covariates in the model in TASSEL software. For identifying significant marker‐trait associations, the threshold *p*‐value of .03 was estimated and used for all traits according to Chan et al. (2010) and Pasam et al. (2012).

## 3 RESULTS

### 3.1 Phenotypic variation

High levels of variation were observed among genotypes for studied traits according to ANOVA. The variance analysis showed that the effect of the environment was significant on some studied traits such as proline content (PRO), water‐soluble carbohydrate concentration (WSC), catalase (CAT), guaiacol peroxidase (POD), and ascorbate peroxidase (APOX). The effect of genotype (G), genotype × year (G × Y), genotype × environment (G × E), and genotype × environment ×year (G × E×Y) were significant on all studied traits (Table 1). Heritability of catalase (CAT), guaiacol peroxidase (POD) and ascorbate peroxidase (APOX) was high. Concerning chlorophyll a/b ratio (chl a/b), chlorophyll b (chl b), relative chlorophyll content (RCC), and canopy temperature (CT), the heritability was medium. Whereas for relative water content (RWC), proline content (PRO), water‐soluble carbohydrate concentration (WSC), PSII, and chlorophyll a + b low heritability was observed (Table 2). A positive correlation was observed among chl a, chl b, chl (a + b), and CAR in both water treatment conditions. Proline content was showed a high correlation with chl a, chl b, chl (a + b), and CAR. In well‐watered conditions, chl a, chl b, chl (a + b), and CAR had a significant and positive correlation with APOX but in drought states, they had a significant and negative correlation with APOX. In drought state, a significant and negative correlation and significant and positive correlation was observed between POD and CT and between POD and CAT, respectively. In well‐watered conditions, RWC was not significantly correlated with other traits except POD (Table 3).

**TABLE 1:** Mean squares of studied traits in spring barley (*Hordeum vulgare* L.) under drought stress

Source of variation	CT	RWC	PRO	WSC	RCC	PSII	Chl a	Chl b	Chl (a + b)	Chl a/b	Car	CAT	POD	APOX
Genotype	358.65**	518.23**	3,273.44**	3,203.98**	382.83**	410.18**	387.70**	655.67**	476.74**	447.51**	416.91**	15,368.91**	31,793.93**	16,671.34**
Year	11.47**	20.12**	48.62**	346.20**	1.25	182.33**	10.26**	26.78**	20.09**	7.61**	3.92^*^	15.17**	55.96**	316.77**
Environment	424.52**	0.00	89.02**	62.29**	0.87	215.92**	2.07	0.10	0.67	0.71	0.05	237.30**	155.23**	305.63**
G × Y	334.07**	477.43**	2,877.79**	2,858.08**	605.04**	337.90**	350.09**	568.17**	416.18**	384.16**	342.54**	3,139.29**	2,970.88**	3,139.47**
G × E	476.43**	384.50**	2,582.56**	2,462.62**	267.46**	309.91**	313.64**	476.25**	350.89**	434.78**	272.75**	15,469.49**	12,737.19**	14,159.15**
Y × E	0.001	1.59	154.68**	50.52**	0.11	0.62	2.62	5.28^*^	4.54^*^	0.87	0.07	29.40**	64.88**	39.79**
G × Y × E	374.03**	401.25**	3,205.17**	2,761.74**	237.74**	479.27**	447.29**	539.87**	484.96**	419.67**	372.46**	2,928.29**	2,342.89**	2,390.34**

Abbreviations: APOX, Ascorbate peroxidase activity; Car, Carotenoid content; CAT, Catalase activity; Chl a, Chlorophyll a; Chl a/b, The chlorophyll a/b ratio; Chl b, Chlorophyll b; CT, Canopy temperature; POD, Guaiacol peroxidase activity; PRO, Proline content; PSII, Maximum quantum efficiency of PSII photochemistry (Fv/Fm); RCC, Relative chlorophyll content; RWC, Relative water content; WSC, Water‐soluble carbohydrate concentration.

** Significant at .01 level

**TABLE 2:** Descriptive statistics and heritability (h^2^) for studied physiological traits in spring barley (*Hordeum vulgare* L.) under well‐watered (W) and drought stress (D) conditions across two years

Trait	Conditions	Year	Minimum	Maximum	Mean	Broad sense heritability (%)
CT	W	2016	8.07	15.83	12.07	0.29 ± 0.28
		2017	3.92	17.65	10.04	
	D	2016	17.46	52.20	24.49	0.37 ± 0.22
		2017	10.95	55.92	22.43	
RWC	W	2016	39.17	91.46	72.10	0
		2017	60.70	99.55	83.18	
	D	2016	44.62	94.18	74.74	0.13 ± 0.36
		2017	52.55	97.85	81.28	
PRO	W	2016	0.27	1.10	0.56	0.11 ± 0.33
		2017	0.31	1.36	0.63	
	D	2016	0.29	1.80	0.85	0
		2017	0.33	1.25	0.59	
WSC	W	2016	4.95	18.27	9.78	0.16 ± 0.31
		2017	10.46	31.63	20.005	
	D	2016	5.94	17.39	10.48	0.037 ± 0.38
		2017	12.48	81.39	32.10	
RCC	W	2016	37.08	55.46	46.09	0.22 ± 0.31
		2017	35.50	57.26	45.45	
	D	2016	29.06	65.36	47.12	0.44 ± 0.20
		2017	27.76	62.16	45.96	
PSII	W	2016	0.49	0.69	0.61	0
		2017	0.41	0.65	0.54	
	D	2016	0.54	0.78	0.68	0.027 ± 0.42
		2017	0.47	0.70	0.62	
Chl a	W	2016	0.89	4.25	2.55	0
		2017	0.61	3.11	1.82	
	D	2016	1.13	5.43	2.52	0
		2017	1.001	3.20	2.28	
Chl b	W	2016	0.74	2.36	1.54	0.38 ± 0.24
		2017	0.30	1.26	0.77	
	D	2016	0.48	2.30	1.27	0
		2017	0.46	1.60	0.98	
Chl (a + b)	W	2016	2.04	6.06	4.09	0.10 ± 0.38
		2017	1.06	4.36	2.60	
	D	2016	1.63	7.74	3.79	0
		2017	1.56	4.71	3.26	
Chl a/b	W	2016	0.77	3.77	1.73	0.32 ± 0.32
		2017	1.34	3.56	2.34	
	D	2016	1.56	3.94	2.02	0
		2017	1.60	2.77	2.32	
Car	W	2016	0.16	1.29	0.71	0
		2017	0.16	0.63	0.35	
	D	2016	0.25	1.28	0.70	0
		2017	0.22	0.73	0.43	
CAT	W	2016	0.24	10.07	1.16	0.90 ± 0.016
		2017	0.26	6.09	1.53	
	D	2016	0.15	6.51	0.77	0.91 ± 0.016
		2017	0.13	2.46	0.71	
POD	W	2016	8.68	44.06	23.09	0.95 ± 0.007
		2017	6.01	55.69	22.81	
	D	2016	8.86	58.39	25.28	0.93 ± 0.10
		2017	6.60	66.50	33.03	
APOX	W	2016	1.73	10.20	4.57	0.92 ± 0.014
		2017	2.44	21.48	7.19	
	D	2016	0.80	8.64	3.35	0.91 ± 0.015

Abbreviations: APOX, Ascorbate peroxidase activity; Car, Carotenoid content; CAT, Catalase activity; Chl a, Chlorophyll a; Chl a/b, The chlorophyll a/b ratio; Chl b, Chlorophyll b; CT, Canopy temperature; D, drought stress conditions; POD, Guaiacol peroxidase activity; PRO, Proline content; PSII, Maximum quantum efficiency of PSII photochemistry (Fv/Fm); RCC, Relative chlorophyll content; RWC, Relative water content; W, well‐watered conditions; WSC, Water‐soluble carbohydrate concentration.

**TABLE 3:** Correlation coefficients among studied physiological traits in spring barley (*Hordeum vulgare* L.) under well‐watered (W) and drought stress (D) conditions across two years

Trait	CT	RWC	PRO	WSC	RCC	PSII	Chl a	Chl b	Chl (a + b)	Chl a/b	Car	CAT	POD	APOX
CT		−0.037	−0.030	0.187*	0.104	0.132	0.010	−0.008	0.004	0.027	0.108	0.063	−0.223^**^	0.119
RWC	−0.113		0.064	−0.141	−0.081	−0.112	0.036	0.018	0.029	0.026	0.103	−0.084	−0.143	−0.135
PRO	0.061	−0.052		0.016	0.248^**^	0.159	0.226^**^	0.212^**^	0.224^**^	−0.005	0.248^**^	0.109	−0.062	−0.005
WSC	0.054	−0.136	0.235^**^		0.142	−0.025	−0.081	−0.040	−0.067	−0.029	−0.004	0.038	−0.153	0.015
RCC	−0.082	−0.123	0.013	−0.051		0.205*	0.188*	0.228^**^	0.204*	−0.149	0.147	0.087	0.018	−0.031
PSII	−0.181*	0.014	0.117	0.192*	0.103		0.091	0.091	0.092	0.009	0.110	0.0001	−0.040	0.055
Chl a	−0.060	−0.154	0.177*	−0.085	0.096	0.157		0.932^**^	0.922^**^	−0.170*	0.860^**^	−0.028	0.005	−0.190*
Chl b	−0.027	0.009	0.153	−0.052	0.119	0.119	0.487^**^		0,970^**^	−0.487^**^	0.768^**^	−0.021	0.004	−0.206*
Chl (a + b)	−0.047	−0.099	0.194*	−0.086	0.119	0.159*	0.900^**^	0.817^**^		−0.284^**^	0.841^**^	−0.025	0.005	−0.199*
Chl a/b	−0.001	−0.105	−0.055	−0.038	−0.064	−0.078	0.123	−0.770^**^	−0.299^**^		−0.034	−0.002	−0.023	0.038
Car	−0.040	−0.047	0.110	−0.080	0.056	0.138	0.875^**^	0.422^**^	0.788^**^	0.134		−0.073	−0.125	−0.162*
CAT	0.049	0.130	0.032	−0.065	0.298^**^	0.096	0.088	0.125	0.122	−0.085	0.124		0.214^**^	0.139
POD	0.138	−0.249^**^	0.109	0.005	0.192*	−0.004	0.283^**^	0.214^**^	0.294^**^	−0.033	0.191*	−0.035		0.151
APOX	0.034	−0.127	0.029	0.043	0.017	0.180*	0.245^**^	0.166*	0.239^**^	−0.011	0.171*	0.041	0.124	

Abbreviations: APOX, Ascorbate peroxidase activity; Car, Carotenoid content; CAT, Catalase activity; Chl a, Chlorophyll a; Chl a/b, The chlorophyll a/b ratio; Chl b, Chlorophyll b; CT, Canopy temperature; D, drought stress conditions; POD, Guaiacol peroxidase activity; PRO, Proline content; PSII, Maximum quantum efficiency of PSII photochemistry (Fv/Fm); RCC, Relative chlorophyll content; RWC, Relative water content; W, well‐watered conditions; WSC, Water‐soluble carbohydrate concentration.

Values above the diagonal are correlation coefficients among traits under drought stress conditions; values below the diagonal are correlation coefficients among traits under well‐watered conditions.

* , ^**^Significant at .05 and .01 probability level, respectively.

### 3.2 Population structure

**FIGURE 1:**
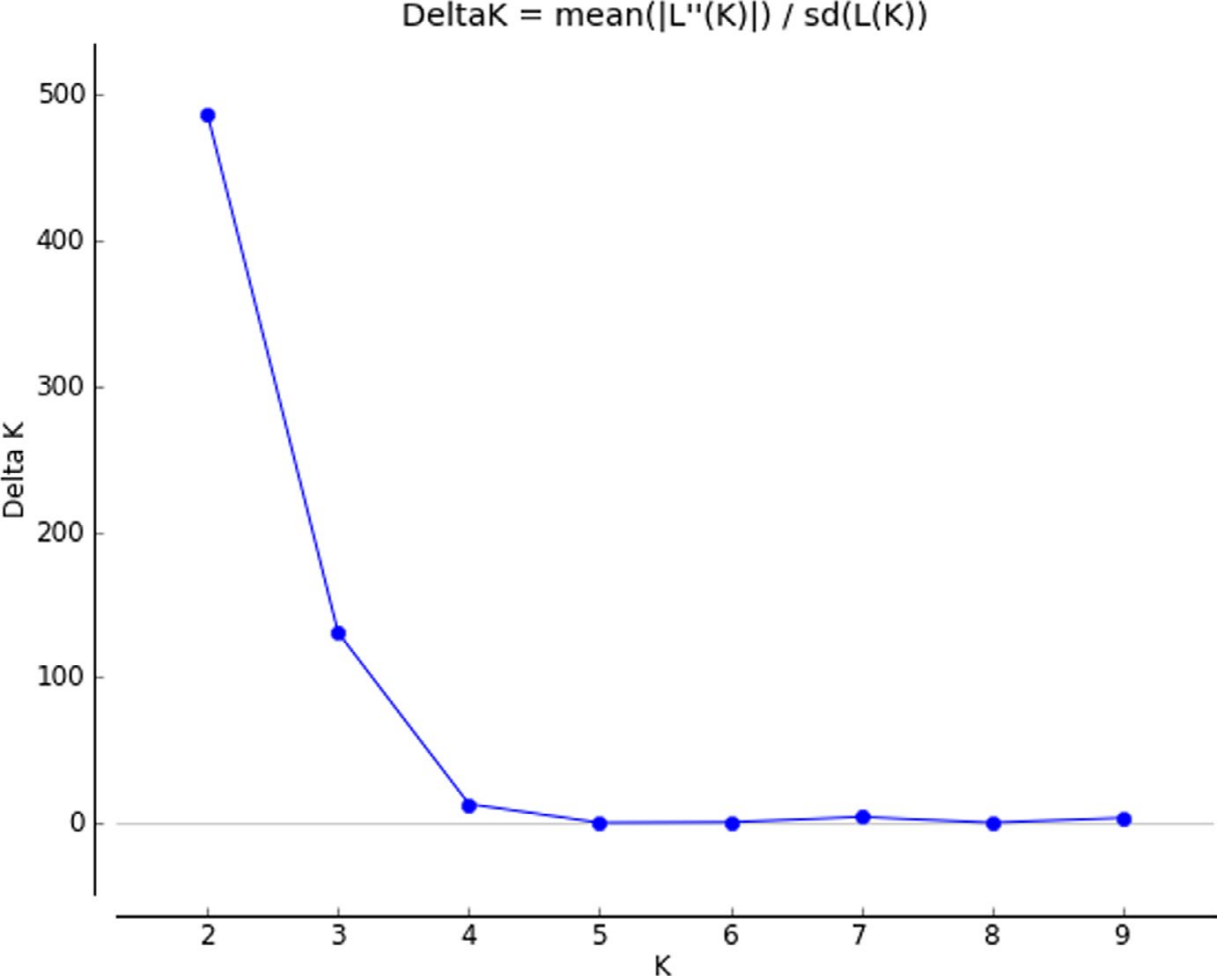
Structure Harvester results using studied DNA markers in spring barley (*Hordeum vulgare* L.)

**FIGURE 2:**
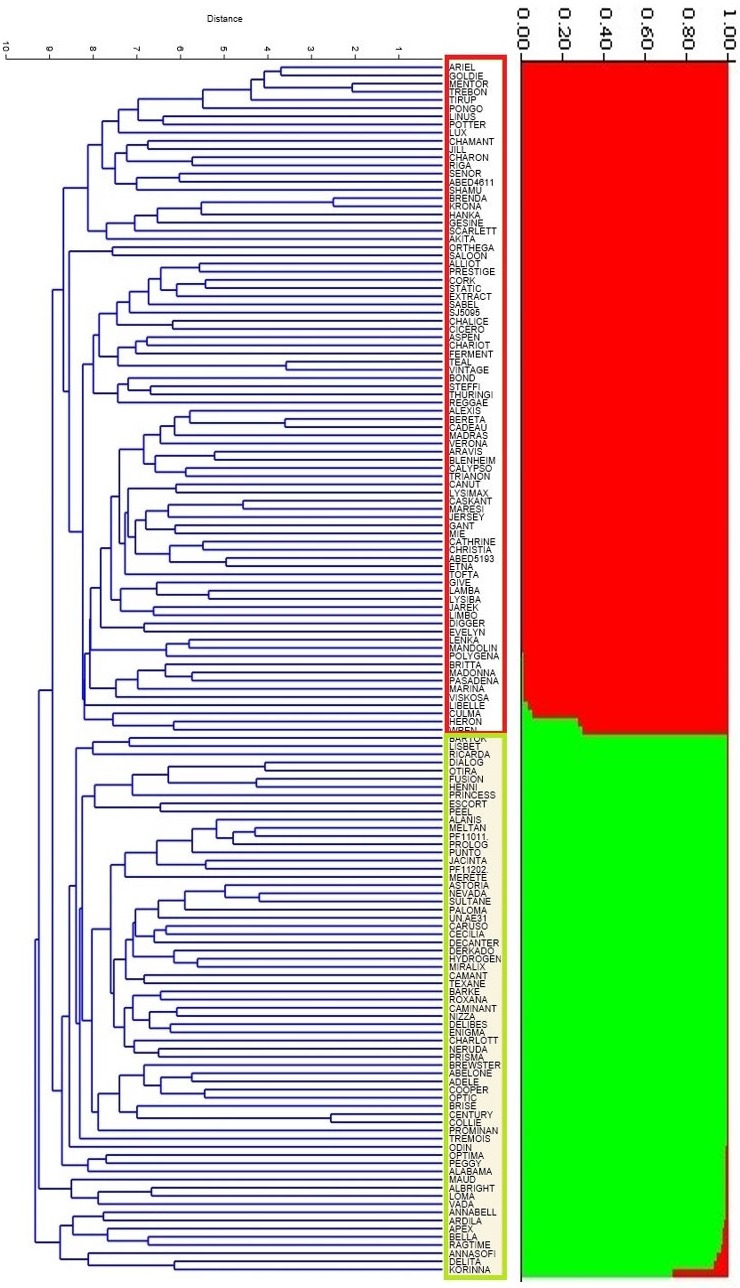
The UPGMA clustering of the accessions based on their genetic distances and the bar plot result according to the structure analysis, both dividing the panel into 2 distinct subgroups; K1 and K2

The genetic fingerprint of 148 barley genotypes was investigated using 407 AFLP and SSR markers. In diagrams of ΔK determined by the Bayesian approach in Structure software 2.3 (Pritchard et al. 2000), the highest value was 2 (Figure 1), which represents that there are two subgroups in this population. Based on *Q* values and membership threshold of 0.7, 83 barley cultivars were assigned to population 1 and 67 barley cultivars were assigned to population 2 (Figure 2).

### 3.3 Linkage disequilibrium and association mapping

**FIGURE 3:**
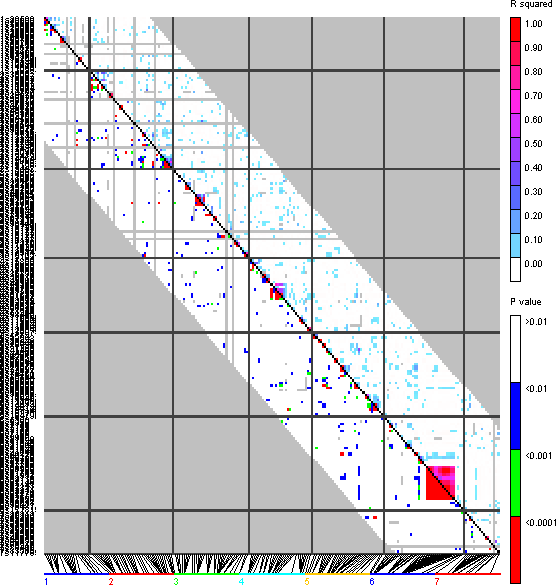
Linkage disequilibrium in spring barley population. The r^2^ values are shown as colored squares above the black diagonal for all polymorphisms with a MAF ≥ 0.05. Colored squares below the black diagonal reflect the significant P‐value

Linkage disequilibrium analysis was performed using molecular markers on the association panel. The *r* ^2^ values among all possible marker pairs showed an average value of 0.0178 (Figure 3). A mixed‐linear model (MLM) method using Q‐ and K‐matrices as covariates were conducted for identifying molecular markers associated with genes controlling physiological traits under well‐watered and drought stress conditions. Results showed the significant association of 207 AFLP and SSR markers with genomic region controlling the fourteen studied traits. In this study, 22 molecular markers were found to be significantly associated with CT from which 11 markers were associated with the trait in well‐watered conditions and the other was associated with the trait in drought stress conditions. From marker associated with the trait in well‐watered conditions, the location of two markers was on linkage group 2H (2016 and 2017) and three were on linkage groups 1H, 6H, and 4H. The location of others was unknown. In drought stress conditions, the location of all identified markers was unknown except to two markers located on linkage groups 2H and 1H. (Table 4).

**TABLE 4:** Association analysis for canopy temperature (CT) and relative water content (RWC) in spring barley (*Hordeum vulgare* L.) under well‐watered and drought stress conditions

Traits	Conditions	Year	Marker	Linkage group	Position (cM)	−Log (P)	*R* ^2^	QTL
CT	W	2016	E45M49‐285	Unknown	–	2.45	.060	–
			E38M50‐334	Unknown	–	1.95	.051	–
			E39M61‐247	1H	95.263	1.76	.039	*W1Q1CT1H*
			Bmac0316‐142	6H	7.155	1.75	.039	*W1Q2CT6H*
			E42M48‐405	2H	150.062	1.64	.036	*W1Q3CT2H*
			E35M54‐078	Unknown	–	1.60	.035	–
			E35M55‐436	Unknown	–	1.59	.036	–
		2017	E45M61‐378	2H	3.800	2.57	.065	*W2Q1CT2H*
			E38M50‐308	Unknown	–	2.17	.057	–
			E37M33‐189	4H	45.305	1.66	.037	*W2Q2CT4H*
			E45M55‐154	Unknown	–	1.51	.033	–
	D	2016	E42M32‐251	Unknown	–	1.88	.044	–
			E42M32‐178	Unknown	–	1.84	.043	–
			E42M32‐176	Unknown	–	1.82	.042	–
			E38M50‐263	Unknown	–	1.52	.033	–
		2017	E37M33‐134	Unknown	–	2.31	.060	–
			E37M33‐260	Unknown	–	2.21	.056	–
			E38M54‐294	2H	151.984	2.18	.052	*D2Q1CT2H*
			E38M54‐127	Unknown	–	2.12	.050	–
			E37M33‐256	Unknown	–	1.76	.042	–
			E37M33‐218	Unknown	–	1.70	.038	–
			E38M54‐260	1H	43.686	1.64	.036	*D2Q2CT1H*
RWC	W	2016	E42M32‐254	Unknown	–	2.11	.052	–
			Bmac0134‐142	2H	10.867	2.06	.051	*W1Q1RWC2H*
			E45M49‐255	Unknown	–	2.05	.048	–
			E35M54‐180	7H	140.172	1.83	.041	*W1Q2RWC7H*
			E35M54‐183	7H	140.172	1.77	.039	*W1Q3RWC7H*
			E37M33‐501	2H	140.642	1.74	.040	*W1Q4RWC2H*
			E33M54‐230	2H	134.720	1.61	.035	*W1Q5RWC2H*
		2017	E38M50‐336	Unknown	–	3.58	.098	–
			E38M50‐284	Unknown	–	3.17	.083	–
			E38M50‐334	Unknown	–	1.63	.038	–
	D	2016	E35M48‐111	Unknown	–	2.28	.084	–
			E35M48‐143	Unknown	–	2.22	.085	–
		2017	E38M55‐089	Unknown	–	3.16	.095	–
			E38M55‐090	Unknown	–	3.16	.095	–
			E35M54‐152	5H	100.372	1.69	.042	*D2Q1RWC5H*
			E38M50‐385	Unknown	–	1.58	.040	–
			E37M33‐237	Unknown	–	1.55	.038	–

Abbreviations: cM, Centimorgan; CT, Canopy temperature; D, drought stress conditions; QTL, Quantitative trait loci; R^2^, Coefficient of determination;RWC, Relative water content; W, well‐watered conditions.

Seventeen markers were found to be significantly associated with RWC from which 10 markers were associated with the trait in well‐watered conditions and the rest 7 markers were associated with the trait in drought stress conditions. The location of 11 out of 17 identified markers was unknown. The location of three markers, identified for the trait under well‐watered conditions, were on the 2H linkage group but in different parts, and the location of two markers was on linkage group 7H in the same region (140.172). The location of one marker identified for the trait under drought stress conditions was on linkage group 5H (Table 4).

Twelve markers showed significant associations with PRO; 10 associated with trait under normal conditions and the rest 2 with that under drought stress conditions. From identified QTLs for the trait under normal conditions, two QTLs are located on linkage group 7H but in different positions and four are located on linkage groups 6H, 4H, 5H, and 2H. From identified QTLs for the trait under drought stress conditions, the location of one marker was on linkage group 5H. The location of four markers associated with traits under normal conditions and one marker with traits under drought stress conditions were unknown (Table 5).

**TABLE 5:** Association analysis for proline (PRO) and water‐soluble carbohydrate concentration (WSC) in spring barley (*Hordeum vulgare* L.) under well‐watered and drought stress conditions

Traits	Conditions	Year	Marker	Linkage group	Position (cM)	−Log (P)	*R* ^2^	QTL
PRO	W	2016	E35M61‐355	Unknown	–	2.71	.071	–
			E42M32‐271	Unknown	–	1.76	.040	–
			E39M61‐255	7H	125.104	1.66	.038	*W1Q1PRO7H*
			E42M32‐243	Unknown	–	1.65	.037	–
			Bmac0316‐170	6H	7.155	1.63	.037	*W1Q2PRO6H*
			E37M33‐311	7H	70.317	1.62	.036	*W1Q3PRO7H*
			E38M54‐144	4H	17.762	1.55	.033	*W1Q4PRO4H*
		2017	E35M48‐410	5H	184.70	1.78	.040	*W2Q1PRO5H*
			E358M48‐411	Unknown	–	1.78	.040	–
			Bmac0134‐142	2H	10.867	1.69	.042	*W2Q2PRO2H*
	D	2016	–	–	–	–	–	–
		2017	E33M54‐148	Unknown	–	1.69	.042	–
			E42M48‐203	5H	157.148	1.68	.040	*D2Q1PRO5H*
WSC	W	2016	E42M32‐304	6H	104.871	1.84	.043	*W1Q1WSC6H*
			E37M33‐372	Unknown	–	1.78	.041	–
			E35M55‐436	Unknown	–	1.71	.039	–
			E35M55‐434	Unknown	–	1.69	.037	–
			E45M49‐285	Unknown	–	1.54	.033	–
			E37M33‐191	4H	45.305	1.50	.032	*W1Q2WSC4H*
		2017	E39M61‐247	1H	95.263	2.39	.059	*W2Q1WSC1H*
			E45M49‐285	Unknown	–	2.23	.053	–
			E38M50‐284	Unknown	–	2.005	.047	–
			E38M50‐336	Unknown	–	1.83	.044	–
			E38M50‐242	Unknown	–	1.81	.043	–
			E42M48‐376	2H	154.422	1.76	.040	*W2Q2WSC2H*
			E35M55‐306	Unknown	–	1.69	.037	–
			E37M33‐226	Unknown	–	1.63	.036	–
			E38M54‐294	2H	151.984	1.59	.035	*W2Q3WSC2H*
	D	2016	E35M55‐436	Unknown	–	2.17	.054	–
			E42M32‐211	Unknown	–	1.80	.043	–
			E38M50‐269	Unknown	–	1.77	.041	–
			E35M55‐434	Unknown	–	1.76	.040	–
			E42M48‐139	4H	63.099	1.72	.039	*D1Q1WSC4H*
			E42M32‐304	6H	104.871	1.63	.037	*D1Q2WSC6H*
		2017	E37M33‐93	3H	126.421	1.51	.040	*D2Q1WSC3H*

Abbreviations: cM: Centimorgan; D, drought stress conditions; PRO, Proline content; QTL: Quantitative trait loci; R^2^: Coefficient of determination; W, well‐watered conditions; WSC, Water‐soluble carbohydrate concentration.

Twenty‐two markers were found to be significantly associated with WSC, from which 15 markers associated with trait under normal conditions and the rest 7 markers associated with that under drought stress conditions. From markers identified for the trait under normal conditions, two markers were from linkage group 2H but in different positions and the three markers were located on linkage groups 6H, 4H, and 1H. The location of 10 out of 15 markers under normal conditions was unknown. From 7 markers identified for the trait under drought stress conditions, three markers were located on linkage groups 4H, 6H, and 3H but the location of four were unknowns (Table 5).

Fourteen markers were found to be significantly associated with RCC, from which 10 markers linked with gene controlling trait under normal conditions and four were associated with genes controlling traits under drought stress conditions. From identified markers for the trait under normal state, the location of two markers was on linkage group 2H but in different regions and the location of three markers was on linkage groups 3H, 4H, and 1H. The location of five out of 10 markers was unknown. Concerning identified markers for the trait under drought stress conditions, the location of two markers was on linkage group 1H but in different positions and the location of two markers was on linkage groups 3H and 4H (Table 6).

**TABLE 6:** Association analysis for relative chlorophyll content (RCC) and the maximum quantum efficiency of PSII photochemistry (Fv/Fm); in spring barley (*Hordeum vulgare* L.) under well‐watered and drought stress conditions

Traits	Conditions	Year	Marker	Linkage group	Position (cM)	−Log (P)	*R* ^2^	QTL
RCC	W	2016	E38M50‐242	Unknown	–	1.98	.046	–
			E37M33‐583	3H	11.996	1.94	.047	*W1Q1RCC3H*
			E35M55‐262	4H	120.643	1.80	.041	*W1Q2RCC4H*
			E38M50‐119	Unknown	–	1.79	.042	–
			HVM54‐158	2H	122.406	1.51	.034	*W1Q3RCC2H*
			Bmac0134‐173	2H	10.867	1.50	.033	*W1Q4RCC2H*
			E45M49‐144	Unknown	–	1.50	.034	–
		2017	E45M49‐285	Unknown	–	2.10	.049	–
			E39M61‐247	1H	95.263	1.85	.044	*W2Q1RCC1H*
			E38M50‐094	Unknown	–	1.67	.036	–
	D	2016	E39M61‐160	1H	44.900	1.64	.042	*D1Q1RCC1H*
		2017	E37M33‐83	4H	116.987	2.42	.067	*D2Q1RCC4H*
			E45M55‐212	3H	98.496	1.66	.037	*D2Q2RCC3H*
			E38M54‐367	1H	51.941	1.65	.037	*D2Q3RCC1H*
PSII	W	2016	E35M54‐152	5H	100.372	2.32	.060	*W1Q1PSII5H*
			E39M61‐106	5H	44.900	1.88	.048	*W1Q2PSII5H*
			E42M48‐282	5H	114.402	1.51	.036	*W1Q3PSII5H*
		2017	Bmac0134‐173	2H	10.867	1.53	.038	*W2Q1PSII2H*
	D	2016	E38M54‐168	Unknown	–	1.91	.045	–
			E35M48‐380	Unknown	–	1.86	.043	–
			E39M61‐082	Unknown	–	1.81	.043	–
			E38M50‐269	Unknown	–	1.69	.038	–
			E37M33‐152	Unknown	–	1.68	.039	–
		2017	E33M54‐421	Unknown	–	2.24	.060	–
			E39M61‐082	Unknown	–	1.93	.047	–
			E38M50‐242	Unknown	–	1.88	.043	–
			E45M49‐255	Unknown	–	1.68	.037	–
			E35M54‐152	5H	100.372	1.52	.033	*D2Q1PSII5H*

Abbreviations: cM, Centimorgan; D, drought stress conditions; PSII, The maximum potential quantum efficiency of PSII photochemistry (Fv/Fm); QTL, Quantitative trait loci; R^2^, Coefficient of determination;RCC, Relative chlorophyll content; W, well‐watered conditions.

Concerning to PSII trait, a total of 14 markers were identified from which four markers were associated with trait under normal conditions and the rest 10 markers associated with trait under drought stress conditions. From identified markers for the trait under normal conditions, the location of all markers was clear and known; one was on linkage group 2H and three were on linkage group 5H but in different positions. From identified markers for the trait under drought stress conditions, the location of all markers except one was unknown (Table 6).

Twelve markers showed significant associations with chl a, from which seven markers associated with trait under normal conditions and the rest five markers associated with trait under drought stress conditions. The location of five out of seven markers was known, on linkage groups 2H, 4H, and 6H. The location of the two was unknown. Concerning drought stress conditions, just the location of one marker was clear and known and the location of rest markers was unknown. The marker with a known location was from linkage group 6H (Table 7).

**TABLE 7:** Association analysis for chlorophyll a and chlorophyll b content in spring barley (*Hordeum vulgare* L.) under well‐watered and drought stress conditions

Traits	Condition	Year	Marker	Linkage group	Position (cM)	−Log (P)	*R* ^2^	QTL
Chl a	W	2016	E35M55‐302	4H	55.763	1.58	.039	*W1Q1Chla4H*
			E37M33‐501	2H	140.642	1.57	.040	*W1Q2Chla2H*
		2017	E42M48‐380	6H	121.819	2.15	.055	*W2Q1Chla6H*
			E38M55‐139	4H	68.628	2.05	.052	*W2Q2Chla4H*
			E38M54‐169	2H	58.579	1.80	.042	*W2Q3Chla2H*
			E35M48‐170	Unknown	–	1.69	.037	–
			E37M33‐256	Unknown	–	1.53	.034	–
	D	2016	Bmac0316‐142	6H	7.155	1.63	.041	*D1Q1Chla6H*
		2017	E35M48‐384	Unknown	–	2.57	.067	–
			E38M54‐245	Unknown	–	2.30	.055	–
			E38M50‐120	Unknown	–	1.95	.048	–
			E38M50‐134	Unknown	–	1.65	.040	–
Chl b	W	2016	E42M32‐200	5H	76.744	1.83	.043	*W1Q1Chlb5H*
			E45M49‐068	Unknown	–	1.60	.035	–
		2017	E38M55‐139	4H	68.628	2.16	.054	*W2Q1Chlb4H*
			E38M54‐169	2H	58.579	2.15	.052	*W2Q2Chlb2H*
			E42M48‐380	6H	121.819	2.08	.052	*W2Q3Chlb6H*
			E38M54‐168	Unknown	–	1.84	.043	–
			E37M33‐256	Unknown	–	1.77	.041	–
			E37M33‐93	3H	126.421	1.50	.033	*W2Q4Chlb3H*
	D	2016	–	–	–	‐	–	–
		2017	E35M48‐384	Unknown	–	2.88	0.076	–
			E38M54‐245	Unknown	–	2.75	0.069	–
			E45M55‐349	7H	133.136	1.96	0.049	*D2Q1Chlb7H*
			E38M50‐120	Unknown	–	1.95	.048	–
			E38M50‐134	Unknown	–	1.68	.040	–
			E35M48‐380	Unknown	–	1.62	.037	–

Abbreviations: Chl a, chlorophyll a; Chl b, chlorophyll b; cM, Centimorgan; D, drought stress conditions; QTL, Quantitative trait loci; R^2^, Coefficient of determination;W, well‐watered conditions.

Concerning chl b, eight markers were identified as significantly associated with genes controlling trait under normal conditions. The locations of five markers were on linkage groups 5H, 4H, 2H, 6H, and 3H. The locations of the three markers were unknown. Six markers associated with genes controlling trait under drought stress conditions. The location of one marker was on linkage group 7H, and the location of the other was unknown (Table 7).

Totally 11 DNA markers were identified for chl (a + b), from which seven markers associated with trait under normal conditions and the rest four markers associated with trait under drought stress conditions. The location of five markers identified for the trait under normal conditions was on linkage groups 3H, 5H, 4H, 6H, and 2H and the locations of two markers were unknown. The location of markers identified for the trait under drought stress conditions was unknown (Table 8).

**TABLE 8:** Association analysis for the total chlorophyll (a + b) content, the chlorophyll a/b ratio, and Carotenoid in spring barley (*Hordeum vulgare* L.) under well‐watered and drought stress conditions

Traits	Conditions	Year	Marker	Linkage group	Position (cM)	−Log (P)	*R* ^2^	QTL
Chl (a + b)	W	2016	E42M32‐118	3H	151.031	1.68	.041	*W1Q1TChl3H*
			E42M32‐200	5H	76.744	1.62	.036	*W1Q2TChl5H*
		2017	E38M55‐139	4H	68.628	2.14	.055	*W2Q1TChl4H*
			E42M48‐380	6H	121.819	2.08	.053	*W2Q2TChl6H*
			E38M54‐169	2H	58.579	1.92	.045	*W2Q3TChl2H*
			E35M48‐170	Unknown	–	1.70	.038	–
			E37M33‐256	Unknown	–	1.56	.035	–
	D	2016	–	–	–	‐	‐	–
		2017	E35M48‐384	Unknown	–	2.63	.068	–
			E38M54‐245	Unknown	–	2.38	.058	–
			E38M50‐120	Unknown	–	1.89	.046	–
			E38M50‐134	Unknown	–	1.62	.038	–
Chl a/b	W	2016	Bmag0223‐156	5H	86.880	1.65	.040	*W1Q1ab5H*
		2017	E38M54‐168	Unknown	–	1.97	.054	–
			E38M50‐414	Unknown	–	1.56	.038	–
	D	2016	E42M32‐069	Unknown	–	1.93	.059	–
			E42M32‐250	5H	130.999	1.63	.045	*D1Q1ab5H*
			E38M54‐169	2H	58.579	1.61	.044	*D1Q2ab2H*
		2017	E37M331‐34	Unknown	–	1.84	.043	–
			E38M54‐133	4H	125.075	1.74	.039	*D2Q1ab4H*
			E38M50‐135	Unknown	–	1.71	.040	–
			E35M55‐262	4H	120.643	1.68	.038	*D2Q2ab4H*
			E45M49‐358	Unknown	–	1.58	.036	–
			E35M54‐180	7H	140.172	1.55	.034	*D2Q3ab7H*
Car	W	2016	E42M32‐118	3H	151.031	1.82	.050	*W1Q1CAR3H*
			E45M49‐382	Unknown	–	1.81	.046	–
			E45M49‐380	Unknown	–	1.77	.045	–
			E42M32‐113	Unknown	–	1.68	.044	–
			E42M32‐115	3H	151.031	1.55	.039	*W1Q2CAR3H*
			E35M55‐302	4H	55.763	1.53	.036	*W1Q3CAR4H*
		2017	E45M49‐254	Unknown	–	2.008	.049	–
			E38M50‐308	Unknown	–	1.81	.044	–
			E42M48‐087	5H	74.760	1.79	.040	*W2Q1CAR5H*
			E38M55‐139	4H	68.628	1.63	.039	*W2Q2CAR4H*
			E38M54‐169	2H	58.579	1.52	.033	*W2Q3CAR2H*
	D	2016	E35M55‐302	4H	55.763	1.53	.037	*D1Q1CAR4H*
		2017	E38M54‐245	Unknown	–	2.35	.057	–
			E38M50‐120	Unknown	–	1.97	.049	–
			E38M50‐242	Unknown	–	1.74	.043	–
			E38M50‐134	Unknown	–	1.68	.041	–
			E35M48‐384	Unknown	–	1.68	.039	–
			E38M50‐119	Unknown	–	1.66	.042	–
			E37M33‐203	Unknown	–	1.52	.036	–

Abbreviations: Car, Carotenoid; Chl a/b, The chlorophyll a/b ratio; cM, Centimorgan; D, drought stress conditions; QTL, Quantitative trait loci; R^2^, Coefficient of determination;W, well‐watered conditions.

For chl a/b, 12 DNA markers were found to be significantly associated with the index. Three out of 12 identified markers associated with the index under normal conditions and the rest (nine markers) associated with the index under drought stress conditions. In normal conditions (well‐watered treatment), the locations of all identified markers were unknown except for one marker on linkage group 5H at region 86.880 cM. In drought stress state, two identified markers were from linkage group 4H but from different regions (125.075 cM and 120.643 Cm), three QTLs were from linkage groups 5H, 2H, and 7H and the location of others were unknown (Table 8).

Nineteen markers were found to be significantly associated with Carotene content (Car); among these 11 markers were associated with genes controlling trait under normal conditions and the rest eight markers associated with genes controlling traits under drought stress conditions. The location of six markers associated with trait under normal conditions was clear and know, on linkage groups 2H, 3H, 4H, and 5H. Indeed, two markers were located on linkage group 3H but in the same location (151.031 cm), two markers located on linkage group 4H and two markers located on linkage groups 5H and 2H. Concerning drought stress conditions, except for one QTL located on linkage group 4H, the location of others was unknown (Table 8).

For CAT, totally, 13 markers were found to be significantly associated with genes controlling trait. Seven out of 13 were found for the trait under normal conditions. The location of one marker is known; on linkage group 2H, the locations of six other markers were unknown. Six markers from 13 identified markers were associated with trait under drought stress conditions. The locations of three were known; on linkage groups, 3H and 5H and the locations of three others were unknown (Table 9).

**TABLE 9:** Association analysis for antioxidant enzymes activity in spring barley (*Hordeum vulgare* L.) under well‐watered and drought stress conditions

Traits	Conditions	Year	Marker	Linkage group	Position (cM)	−Log (P)	*R* ^2^	QTL
CAT	W	2016	E42M32‐254	Unknown	–	2.55	.095	–
		2017	E42M32‐254	Unknown	–	2.97	.081	–
			E38M50‐120	Unknown	–	2.43	.061	–
			E38M50‐134	Unknown	–	2.42	.065	–
			E38M54‐390	2H	88.013	2.34	.057	*W2Q1CAT2H*
			E45M49‐144	Unknown	–	1.82	.044	–
			E33M54‐148	Unknown	–	1.69	.037	–
	D	2016	E35M61‐210	3H	135.142	1.71	.039	*D1Q1CAT3H*
			E45M49‐358	Unknown	–	1.62	.036	–
			E33M54‐421	Unknown	–	1.61	.038	–
			E45M55‐164	Unknown	–	1.50	.033	–
		2017	E35M48‐400	5H	183.752	2.91	.076	*D2Q1CAT5H*
			E37M33‐93	3H	126.421	1.53	.035	*D2Q2CAT3H*
POD	W	2016	E38M50‐456	Unknown	–	2.32	.057	–
			E35M48‐380	Unknown	–	1.86	.044	–
			E45M55‐349	7H	133.136	1.81	.043	*W1Q1POD7H*
		2017	E35M48‐380	Unknown	–	2.36	.058	–
			E38M50‐456	Unknown	–	1.74	.040	–
			Bmac0316‐170	6H	7.155	1.68	.037	*W1Q2POD6H*
			E37M33‐218	Unknown	–	1.54	.035	–
			E35M48‐111	Unknown	–	1.52	.034	–
	D	2016	E45M55‐108	Unknown	–	2.03	.068	–
			E35M48‐170	Unknown	–	1.62	.049	–
		2017	E38M50‐094	Unknown	–	2.03	.048	–
			E35M48‐380	Unknown	–	2.01	.048	–
			E42M32‐200	5H	76.744	1.73	.041	*D2Q1POD5H*
APOX	W	2016	E35M54‐243	2H	11.874	2.39	.058	*W1Q1APOX2H*
			E35M61‐378	2H	3.800	1.67	.040	*W1Q2APOX2H*
			E39M61‐255	7H	125.104	1.55	.034	*W1Q3APOX7H*
			E38M50‐135	Unknown	–	1.53	.035	–
		2017	E39M61‐180	2H	48.823	1.59	.044	*W2Q1APOX2H*
			E39M61‐181	2H	48.890	1.59	.044	*W2Q2APOX2H*
	D	2016	E38M50‐308	Unknown	–	2.32	.063	–
			E35M48‐087	Unknown	–	1.81	.041	–
			E37M33‐218	Unknown	–	1.78	.042	–
			E35M48‐384	Unknown	–	1.76	.040	–
			E37M33‐93	3H	126.421	1.69	.039	*D1Q1APOX3H*
			Bmac0134‐151	2H	10.867	1.63	.037	*D1Q2APOX2H*
		2017	–	–	–	–	–	–

Abbreviations: APOX, Ascorbate Peroxidase; CAT, Catalase; cM, Centimorgan; D, drought stress conditions; POD, Guaiacol Peroxidase; QTL, Quantitative trait loci; R^2^, Coefficient of determination; W, well‐watered conditions.

For POD, 13 DNA markers were identified. The location of all of them was unknown except to two markers on linkage groups 7H and 6H, identified for the trait under normal conditions (well‐watered treatment) and one on linkage group 5H, identified for the trait under drought stress conditions (Table 9).

Totally, 12 DNA markers were found to be significantly associated with the APOX trait; among them the location of five markers was unknown and the locations of others were known; on different linkage groups. From markers identified for the trait under normal conditions, the locations of four QTLs were on linkage group 2H but in different regions and the location of one was on linkage group 7H. Concerning markers identified for the trait under drought stress conditions the locations of two markers were known; on linkage groups 3H and 2H (Table 9).

## 4 DISCUSSION

Population size, degree, of LD and quality of phenotypic data are important factors that affect the success of association studies (Flint‐Garcia et al., 2005; Mackay & Powell, 2007). The results showed high phenotypic diversity for studied traits among barley cultivars. The effect of genotype (G) and genotype and year interaction (G × Y) was significant on all studied traits. The efficiency of phenotypic selection is affected by G × E interaction (Sallam et al., 2019). Then, marker‐assisted selection can be important in such a condition. According to Stansfield (1991), if the heritability of the trait is more than 0.5, the heritability is considered high, if it is between 0.2 to 0.5, the heritability is considered medium and in this case, if it is lower than 0.2, the heritability is considered low. Then, in this study catalase (CAT), guaiacol peroxidase (POD) and ascorbate peroxidase (APOX) had high heritability.

In population structure analysis, the studied association panel was subdivided into 2 subpopulations. Population structure affects the efficiency of association analysis (Sorkheh et al., 2008). If it exists and is not considered in the association model, probability some false‐positive markers will be identified that are not important because of marker‐assisted selection (Pritchard et al., 2000). Barley has a wide level of population structure because of its two‐rowed versus six‐rowed cultivars or spring versus winter barley cultivars (Pasam et al., 2012). Because of the complex population structure, there could be a higher challenge in GWAS than QTL mapping by producing false‐positive markers (Myles et al., 2009; Pasam et al., 2012). The mixed linear model can overcome these false associations (Yu et al., 2006). LD is another critical factor that affects the resolution of association analysis (Remington et al., 2001). The *r* ^2^ values (an index for evaluating the LD extend) showed an average value of 0.0178. Several studies have previously reported various rates of LD in different barley populations (Caldwell et al., 2006; Ramsay et al., 2011; Stracke et al., 2007) and among different chromosomes (Rostoks et al., 2006). Caldwell et al. (2006) reported rapid decay of LD in barley landraces compared to elite barley cultivars.

In this study, a total of 207 DNA markers were identified for studied physiological traits under normal (well‐watered) and drought stress conditions. A few studies presented reports about the identification of QTLs for water‐soluble carbohydrates (Diab et al., 2004; Teulat et al., 2001) and proline content (Fan et al., 2015; Sayed et al., 2012) in barley. Teulat et al. (2001) investigated plant water status in barley under two water regimes and found one QTL related with WSC on linkage group 2H in drought stress conditions and one QTL related with trait on linkage group 4H in irrigated conditions. In our study, QTLs for WSC were found on linkage groups 1H, 2H, 3H, 4H, and 6H in both water treatment conditions. Sayed et al. (2012) reported four QTLs for proline content (PRO) in barley on linkage groups 3H, 4H, 5H, and 6H under drought stress conditions. Siahsar and Aminfar (2010) reported two QTLs for proline accumulation in barley under salt stress conditions on linkage group 5H using 72 double haploid lines of a cross between Steptoe and Morex cultivars. In the present study, the identified QTLs for proline content (PRO) under normal conditions were on linkage groups 2H, 5H, 4H, 6H, and 7H and that identified for the trait under drought stress conditions was on linkage group 5H.

QTLs for RWC were identified on linkage groups 2H and 7H in well‐watered conditions and that identified for the trait under drought stress state was on linkage group 5H. Mohamed et al. (2015) reported QTLs for RWC in barley under the normal condition on linkage groups 1H, 3H, and 6H and QTLs for the trait under salt stress conditions on linkage groups 2H, 3H, 5H, 7H, and 6H. Also, Chen et al. (2010) reported QTLs for RWC in barley under drought stress conditions on linkage group 2H. In the present study, for chlorophyll content, QTLs were identified on linkage groups 1H, 2H, 3H, and 4H under both water treatment conditions. Barati et al. (2017) reported two and four QTLs for chlorophyll content in barley under normal and stress conditions on linkage groups 3H, 4H, 5H, and 6H. For catalase activity under well‐watered treatment, one QTL was identified on linkage group 2H and that for the trait under drought stress conditions was identified on linkage groups 3H and 5H. Gudys et al. (2018) reported one QTL for catalase activity in barley in irrigated conditions on linkage group 2H and they did not detect any QTL for the trait under drought stress conditions.

Some identified DNA markers were common among some evaluated traits. For example, under normal conditions, E45M39‐285 associated with three characters (CT, WSC, and RCC), E38M50‐334 was related with CT and RWC, E42M32‐254 was common between RWC and CAT, Bmac0134‐142 was associated with RWC and PRO, E37M33‐501 was related with RWC and Chl a, and Bmac0134‐173 was related with RCC and PSII. Under drought stress conditions, E38M50‐269 was associated with WSC and PSII, E33M54‐421 was related with PSII and CAT, E35M48‐384 was associated with five characters (Chl a, Chl b, Chl (a + b), Car, APOX), E38M54‐245 was related with four characters (Chl a, Chl b, Chl (a + b), and Car), and E45M49‐358 was associated with Chl a/b and CAT. Traits with common DNA markers had also shown significant correlations at phenotypic levels. For instance, based on correlation analysis of phenotypic data, Chl a, Chl b, and Chl (a + b) had a significant correlation and GWAS analysis showed that E42M48‐380 was common for these traits under normal conditions. The common markers between some of the traits can be due to linkage or pleiotropic effects. The common markers are useful because they lead to an increase in the efficiency of marker‐assisted selection. Some common markers were identified for a particular trait or some traits across normal and drought stress conditions. For example, E42M32‐304 and E35M55‐434 were associated with WSC in normal and drought stress conditions. Bmac0316‐142 was associated with CT under normal and with Chl a under drought stress conditions. E35M55‐436 was related to CT under normal and with WSC under drought stress conditions. E38M50‐308 was associated with CT and Car under normal and with APOX under drought stress conditions. E38M54‐294 was related to WSC under normal and with CT under drought stress conditions. E37M33‐256 was associated with Chl a, Chl b, and Chl (a + b) under normal and with CT under drought stress conditions. E45M49‐255 was related with RWC under normal and with PSII under drought stress conditions. E35M54‐180 was associated with RWC under normal conditions and with Chl a/b under drought stress conditions. E35M48‐111 was associated with RWC under drought stress conditions and with POD in normal conditions. E35M54‐152 was related to PSII in normal and drought stress conditions and also with RWC under drought stress conditions. E38M50‐242 was associated with WSC and RCC under normal conditions and with PSII and Carotene under drought stress conditions. E37M33‐93 was related to Chl b under normal conditions and with WSC, CAT, and APOX under drought stress conditions. E35M55‐262 was associated with RCC under normal conditions and with Chl a/b in drought stress conditions. E38M50‐119 was associated with RCC under normal conditions and with Carotene in drought stress conditions. E38M50‐094 was related to RCC under normal conditions and with POD under drought stress conditions. E35M48‐380 was associated with POD under both normal and drought stress conditions and also with Chl b and PSII under drought stress conditions. E35M55‐302 was related with Carotene under both normal and drought stress conditions and also with Chl a under normal conditions. E35M48‐170 was related with Chl a and Chl (a + b) under normal conditions and with POD under drought stress conditions. E38M50‐134 was associated with CAT under normal conditions and with Chl a, Chl b, and Carotene under drought stress conditions. E38M50‐120 was related with CAT under normal conditions and with Chl b, Chl (a + b), and Carotene under drought stress conditions. E38M50‐135 was associated with APOX under normal conditions and with Chl a/b in drought stress conditions. These markers show low interaction with environmental conditions (stable markers); therefore, selection by them for a trait under normal conditions will improve the trait value under stress conditions, too.

## 5 CONCLUSIONS

Resistance to drought stress is complex. Results of the present study suggest that association analysis is a powerful tool to identify DNA markers for physiological traits in barley. 207 DNA markers showed significant association with regions controlling the studied physiological traits. 120 QTLs out of 207 QTLs were detected for traits under normal conditions and 90 QTLs were detected for traits under drought stress conditions. Identified markers potentially are useful in marker‐assisted breeding programs for selection to drought stress tolerance. Some loci were common for more than one trait. Detecting common QTLs for some physiological traits and enzymes activity under normal and drought stress conditions can facilitate improving high yielding barley cultivars under drought stress‐prone.

## CONFLICT OF INTEREST

The authors declare that they have no competing interests.

## AUTHOR CONTRIBUTION

Investigation, Data curation, Formal analysis: Mitra Jabbari and Zahra Koochakpour; Conceptualization, Methodology, Supervision: Barat Ali Fakheri, Nafiseh Mahdi Nezhad, and Reza Ataei; Resources: Reza Aghnoum; Writing—original draft Mitra Razi; Writing—review and editing: Reza Darvishzadeh.

## Data Availability

The datasets generated during and/or analyzed during the current study are available from the corresponding author on reasonable request.
